# E1 Ubiquitin-Activating Enzyme UBA-1 Plays Multiple Roles throughout *C. elegans* Development

**DOI:** 10.1371/journal.pgen.1000131

**Published:** 2008-07-18

**Authors:** Madhura Kulkarni, Harold E. Smith

**Affiliations:** 1 Department of Cell Biology and Molecular Genetics, University of Maryland, College Park, Maryland, United States of America; 2 Center for Advanced Research in Biotechnology, University of Maryland Biotechnology Institute, Rockville, Maryland, United States of America; University of Pennsylvania School of Medicine, United States of America

## Abstract

Poly-ubiquitination of target proteins typically marks them for destruction via the proteasome and provides an essential mechanism for the dynamic control of protein levels. The E1 ubiquitin-activating enzyme lies at the apex of the ubiquitination cascade, and its activity is necessary for all subsequent steps in the reaction. We have isolated a temperature-sensitive mutation in the *Caenorhabditis elegans uba-1* gene, which encodes the sole E1 enzyme in this organism. Manipulation of UBA-1 activity at different developmental stages reveals a variety of functions for ubiquitination, including novel roles in sperm fertility, control of body size, and sex-specific development. Levels of ubiquitin conjugates are substantially reduced in the mutant, consistent with reduced E1 activity. The *uba-1* mutation causes delays in meiotic progression in the early embryo, a process that is known to be regulated by ubiquitin-mediated proteolysis. The *uba-1* mutation also demonstrates synthetic lethal interactions with alleles of the anaphase-promoting complex, an E3 ubiquitin ligase. The *uba-1* mutation provides a sensitized genetic background for identifying new *in vivo* functions for downstream components of the ubiquitin enzyme cascade, and it is one of the first conditional mutations reported for the essential E1 enzyme in a metazoan animal model.

## Author Summary

Proteins that control an organism's development must first be turned on at the proper time and place, and then turned off when they are no longer needed. One of the “off” signals occurs through the attachment of a small protein, known as ubiquitin, to the target protein, which typically leads to the destruction of the target. Attachment of ubiquitin is controlled by a series of enzymes, the first of which is known as E1. Most organisms have a single gene for the E1 enzyme, and its activity is crucial for the degradation of a wide range of target proteins throughout development. We have identified a temperature-sensitive mutation in the E1 enzyme of the nematode *Caenorhabditis elegans*. By manipulating the growth temperature, we have determined the various functions of E1 at different stages of development. We find that this enzyme controls embryonic and larval development, sperm fertility, and body size. We also characterized sex-specific roles for E1; males exhibit progressive paralysis and defects in the tail, which is used for mating. In addition to the knowledge gained, this mutation provides a means of identifying both the functions of other ubiquitin enzymes during development as well as the target proteins that are marked for destruction.

## Introduction

Post-translational modification of proteins performs a critical role in regulating protein activity, and ubiquitin-mediated proteolysis has emerged as the key player in the control of protein turnover. Ubiquitin, a highly conserved small protein, is covalently attached to a target protein through an enzymatic cascade, and the assembly of a poly-ubiquitin chain typically specifies that protein for rapid degradation via the 26S proteasome [1]. Ubiquitin-mediated proteolysis thus provides an “off” switch for governing the spatial and temporal distribution of proteins that are no longer needed. This mode of regulation is essential for normal cellular processes (e.g., cell cycle progression and differentiation), and defects have been implicated in human diseases such as cancers and neurodegenerative disorders [2],[3].

Ubiquitination of target proteins can also regulate function by mechanisms other than proteasome-mediated degradation. Mono-ubiquitination serves a signal for endocytosis and trafficking of various cell surface proteins, and is also implicated in histone and transcription factor regulation [4]–[6]. The assembly of poly-ubiquitin chains can occur at different lysines within ubiquitin, which promotes different outcomes for the labeled protein. Conjugation at lysine 48 typically leads to proteasomal degradation, while linkage through lysine 63 can modulate protein activities in processes as diverse as nuclear localization, DNA repair, or inclusion formation in neurodegenerative diseases [7]–[9].

A trio of enzymes mediates the attachment of ubiquitin to substrate protein: the E1 ubiquitin-activating enzyme, E2 ubiquitin-conjugating enzyme, and E3 ubiquitin ligase [10]. Repeated cycles of ligation to the initial ubiquitin lead to poly-ubiquitination. Substrate specificity is conferred by the selective binding of individual E3 ligases to one or a few target proteins [11]. Eukaryotes typically possess a single gene encoding the E1-activating enzyme, tens of E2-conjugating enzymes, and as many as several hundred E3 ligases. Some E3 ligases are themselves multi-subunit complexes, in which a substrate recognition subunit specifies the protein targeted for ubiquitination.

*In vivo* roles for ubiquitination in organismal development have been determined primarily through the characterization of specific E3 ligases. In the nematode *Caenorhabditis elegans*, E3 ligases regulate processes as diverse as sex determination, cell cycle progression, and synaptic signaling [12]–[16]. Studies of E2 conjugating enzymes indicate interactions with multiple E3s, as their relative numbers would predict. For example, inactivation of *ubc-2* produces a broader range of phenotypes than inactivation of its known E3 partner *apc-11* [17].

One of the best-characterized functions for ubiquitination and proteasomal degradation in *C. elegans* is the coordination of early events of embryogenesis [18]. The anaphase-promoting complex (APC) is a multi-subunit E3 ligase that is essential for completion of meiosis immediately after fertilization of the oocyte by the sperm [19],[20]. Ubiquitin-mediated proteolysis also plays a role in the degradation of several proteins that are involved in establishment of anterior-posterior (A-P) polarity in the early embryo. These proteins become asymmetrically localized at the first cell division, and failure to degrade these components correlates with developmental defects such as changes in cell fate specification and embryonic lethality. Formation of the A-P axis and progression of the embryonic cell cycle requires the activities of a class of E3 complexes known as Cullin-RING ligases [21]–[27]. Mutations in components of the APC also affect A-P polarity, possibly as a consequence of defects in meiosis [28],[29].

The E1 ubiquitin-activating enzyme lies at the apex of the enzymatic cascade, and manipulation of its activity might provide a crucial entry point for identifying the myriad roles performed by ubiquitin during development. Temperature-sensitive alleles of E1 have been identified in mammalian cell lines as cell cycle mutations that exhibit reduced ubiquitination and degradation of substrate proteins [30],[31]. Similarly, a temperature-sensitive allele of E1 in yeast dramatically reduces ubiquitin conjugation and also leads to cell cycle arrest [32]. Conditional alleles have also been isolated in Drosophila in a screen for suppressors of *hid*-induced apoptosis during eye development [33]. Detailed characterization demonstrated the complexity of ubiquitin regulation in this system. Whereas weak alleles of the E1-encoding *Uba1* gene block apoptosis, strong alleles promote cell cycle arrest and death. Furthermore, these pro-apototic alleles promote non-autonomous proliferation in adjacent cells via elevated levels of Notch signaling.

We report here the isolation of a temperature-sensitive mutation in the *C. elegans uba-1* gene, which encodes the sole E1 enzyme in this organism. Prior results for RNAi of *uba-1* reported maternal sterility and embryonic lethality, with defects in meiotic progression [34]–[36]. The *uba-1(it129)* mutation recapitulates these phenotypes and also reveals several novel functions, including roles in sperm fertility, body size, and sex-specific development. The *uba-1(it129)* mutation reduces *in vivo* levels of ubiquitin conjugates and causes a delay in meiotic progression in the early embryo, consistent with a reduction in E1 activity. The *uba-1(it129)* mutation also demonstrates synthetic lethal interactions with known components of the anaphase-promoting complex and, as such, provides a sensitized genetic background for identifying new *in vivo* functions for other components of the ubiquitin cascade.

## Results

### Phenotypic Characterization

The temperature-sensitive *it129* allele was isolated by Diane Shakes and, on the basis of sperm sterility and larval lethality, was provisionally designated as *spe-32* (S. Ward, pers. comm.). We have determined that *spe-32* is allelic to *uba-1* (see below), the sole E1 ubiquitin-activating enzyme in *C. elegans*, and have adopted the latter gene name for the sake of clarity. Our detailed characterization of *uba-1(it129)* demonstrates a number of phenotypes, some of which are sex-specific, in addition to those mentioned above.

**Figure 1:**
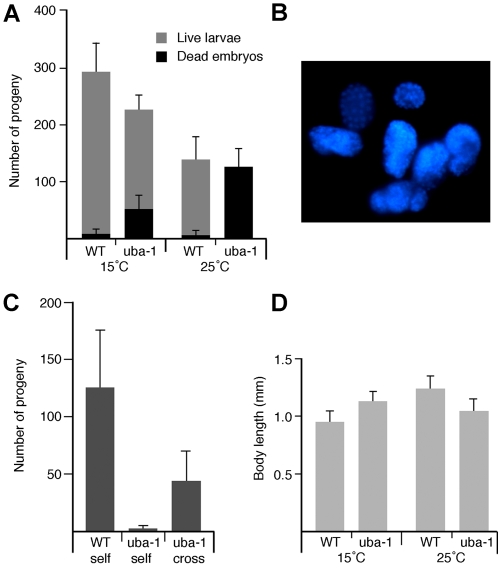
Defects in *uba-1* hermaphrodites. A) Number of viable and inviable progeny produced by wild type (WT) or *uba-1(it129)* hermaphrodites at 15°C or 25°C. Shown are mean values and standard deviations (N = 6) of total progeny. B) DAPI staining of *uba-1(it129)* embryos from adults shifted to 25°C. C) Sperm-specific sterility. Number of viable progeny produced at 25°C by wild type or *uba-1(it129)* hermaphrodites, either unmated (self) or mated with wild-type males (cross). Shown are mean values and standard deviations (N = 6) of progeny produced in 48 h. D) Body length. Mean body length and standard deviations (N = 20) of age-synchronized adult hermaphrodites.

Different phenotypes are manifested at different developmental stages (summarized in Table 1). To facilitate characterization, temperature-shift experiments were performed with age-synchronized populations of *uba-1(it129)* hermaphrodites. Adults shifted to the restrictive temperature produce dead embryos, and the number is equal to the number of progeny produced by wild-type animals at this temperature (Figure 1A). Embryonic arrest is heterogeneous, based on the variable morphology of the embryos and the broad range in the number of nuclei observed with DAPI staining (Figure 1B). Temperature shift at any stage of embryogenesis leads to normal hatching, but 100% of the resulting larvae die at the L2 stage (data not shown). Thus, the *uba-1* gene product is essential for both embryonic and larval development.

**Table 1:** Summary of *uba-1(it129)* phenotypes.

Stage shifted	Phenotype(s)
Adult	F1 embryonic lethality
	Paralysis (male only)
Embryo	Larval lethality
L2/L3 larva	Sperm-specific sterility
	Change in body size
	Tail defect (male only)
	Paralysis (male only)

Larvae that are shifted to the restrictive temperature at the L3 stage exhibit normal somatic development. However, reproduction is adversely affected in the adult hermaphrodite. These sterile animals lay only unfertilized oocytes instead of embryos, but produce viable progeny when mated to wild type males, indicating that the sterility is sperm-specific (Figure 1C). Viability of these outcross progeny is high (96%), suggesting that oocyte development (which occurs subsequent to sperm production in the hermaphrodite) is largely unaffected by the mutation. Detailed characterization of the spermatogenesis defect (described below) indicates that these hermaphrodites produce appropriate numbers of morphologically normal sperm, but that the sperm are incapable of fertilization.

All of the above phenotypes are fully recessive, as heterozygous hermaphrodites are indistinguishable from wild type. These phenotypes are largely though not completely rescued in *uba-1(it129)* homozygous animals at the permissive temperature. There is an increase in embryonic lethality as well as a decrease in the number of embryos produced (Figure 1A), which indicates that the *uba-1(it129)* gene product is not fully functional at 15°C. In addition, temperature causes a small but significant (p<0.001 by Student's t-test) difference in body size between wild-type and *uba-1(it129)* adults (Figure 1D). When reared at 15°C, *uba-1(it129)* hermaphrodites are 16% longer than wild type. The opposite phenotype is observed at 25°C, with the *uba-1(it129)* mutants being 16% shorter than the wild type adults.

In the course of generating heterozygous strains for phenotypic characterization, we observed strong maternal effect rescue of the early developmental defects. Homozygous *uba-1(it129)* progeny derived from +/*uba-1(it129)* hermaphrodites reared at the restrictive temperature exhibited little embryonic or larval lethality (Table 2). Maternal rescue was not complete for all phenotypes; although the homozygous hermaphrodites developed normally into adulthood, sperm-specific sterility was still observed in these animals. We also tested for paternal rescue by mating *uba-1(it129)*/*+* heterozygous males to *uba-1(it129)* homozygous hermaphrodites. Again, embryonic and larval lethality (though not sperm sterility) were largely rescued (Table 2). Because the presence of a single wild-type copy of *uba-1* in either the hermaphrodite or male parent effectively suppresses embryonic and larval lethality in homozygous mutant progeny, it suggests that the maternal or paternal contribution of UBA-1 protein is sufficient to allow somatic development to proceed normally until adulthood.

**Table 2:** Maternal and paternal rescue of lethality.

Rescue	Hermaphrodite genotype	Male genotype	Lethality (predicted)
Maternal	*uba-1(it129)*/+	none (self-fertile)	3.8% (25%)
Paternal	*uba-1(it129)*/*uba-1(it129)*	*uba-1(it129)*/+	7.2% (50%)

Data are from five (maternal) or six (paternal) hermaphrodites. Mean total progeny numbers with s.d. are 123±11 (maternal) and 73±35 (paternal).

### Male-Specific Phenotypes

To facilitate the phenotypic characterization of males, we constructed a *uba-1(it129) him-5(e1490)* strain [the *him-5(e1490)* mutation produces males via nondisjunction of the X chromosome] [37]. Temperature-shift experiments were performed with age-synchronized populations, and the same phenotypes were observed in males as above: embryonic and larval lethality and a reduction in body size (data not shown). Sperm-specific sterility of mutant males was assessed by crossing to *fem-1(hc17)* hermaphrodites, which lack sperm but produce oocytes that can be fertilized by mating. Experiments described below indicate that mating was successful but no cross-progeny were produced, demonstrating that male sperm are incapable of fertilization. Thus, the same array of defects are produced by the *uba-1(it129)* mutation in males and hermaphrodites.

**Figure 2:**
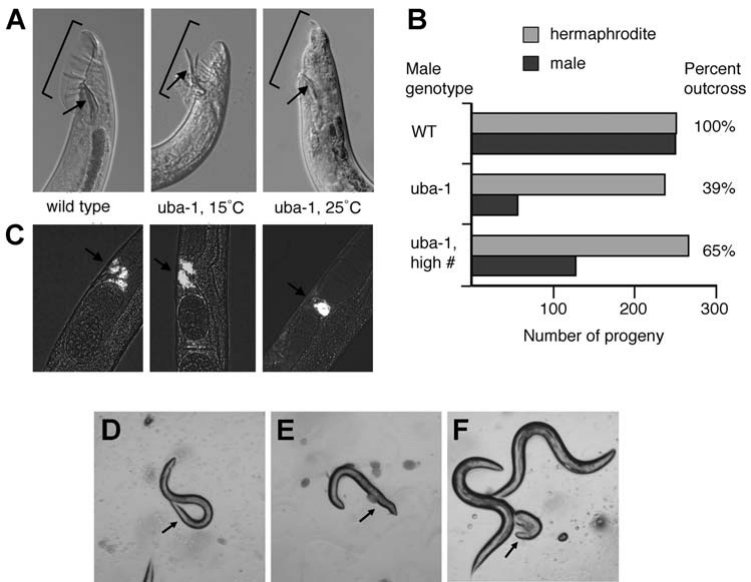
Defects in *uba-1* males. A) Male tail defects. Shown are DIC photomicrographs of the male tail from wild type or *uba-1(it129)* animals grown at the indicated temperature. Bracket indicates the fan and sensory rays. Arrow indicates the spicules. B) Reproductive success of male mating. Graph indicates the mean number of male and hermaphrodite progeny produced by wild type hermaphrodites (N = 6) mated to wild type or *uba-1(it129)* males grown at 15°C. Only the first 500 progeny were counted for wild type. Matings combined one hermaphrodite with five males, or three hermaphrodites with 12 males (high #) for 24 h. Percent outcross is calculated by multiplying the number of male progeny by two, then dividing by the total number of progeny. C) Sperm transfer. Arrow indicates fluorescently-labeled sperm from wild type or *uba-1(it129)* males localized within the spermatheca of unlabeled hermaphrodites after mating. D–F, male-specific paralysis. D) Young adult *uba-1(it129)* male. Arrow indicates normal sinusoidal curve of tail. E) Older adult *uba-1(it129)* male. Arrow indicates flaccid posture of tail. F) Dead *uba-1(it129)* male (arrow) and two *uba-1(it129)* hermaphrodites.

We also observed additional phenotypes in *uba-1(it129)* males. The most conspicuous phenotype in the adult was constitutive protraction of the spicules (Figure 2A, first vs. second panel). These structures are part of the reproductive apparatus of the male tail, and are normally extended only during insertion into the vulva for sperm transfer. A defect in spicule retraction was apparent in adult males at both the permissive and restrictive temperatures. Constitutively protracted spicules were observed in approximately one-third of *uba-1(it129)* males reared at 15°C and nearly all of those reared at 25°C. In some cases the spicules, gubernaculum, and surrounding tissues were everted, suggestive of structural defects in the integrity of the male reproductive tract as well.

Additional abnormalities in the male copulatory apparatus were observed in animals reared at the restrictive temperature. The tail of the wild-type male possesses a cuticular fan containing nine pairs of sensory rays (Figure 2A, first panel), which are involved in mate detection and the behavioral responses necessary for locating the hermaphrodite vulva. The size of the fan is greatly diminished in *uba-1(it129)* homozygous males raised at 25°C, which results in shortening of the tail tip and sensory rays as well (Figure 2A, third panel). The number of rays is not affected, and other male reproductive structures appear superficially normal by light microscopy. The shortened fan phenotype is semi-dominant: the fan expanse in heterozygous *+*/*uba-1(it129)* males is less than in wild-type but greater than in homozygous animals (data not shown). Therefore, proper formation of the male copulatory structures appears to be quite sensitive to the dosage of UBA-1 protein.

The male tail structures are critical for mating behavior and sperm transfer, so aberrations in the fan or in spicule function might adversely affect male reproductive success. Sperm from wild-type males take precedence over hermaphrodite sperm such that only outcross progeny are produced until the male sperm are depleted, at which time the production of self progeny continues [38]. Male sperm produce male and hermaphrodite progeny in equal numbers, while hermaphrodite sperm produce exclusively hermaphrodite progeny. Therefore, the number of outcross progeny, an indicator of male reproductive success, can be readily calculated by determining the number of males produced.

Reproductive success was ascertained for homozygous *uba-1(it129)* males grown at the permissive temperature. Some of these animals have protruding spicules, which might be predicted to impair sperm transfer. The fertility of *uba-1(it129)* hermaphrodites at 15°C demonstrates that sperm function is normal at this temperature, so the production of outcross progeny was used as an indicator of successful mating. Mating to wild-type males produced males and hermaphrodites in the expected 1∶1 ratio, indicating that all of the offspring in the measured time interval resulted from fertilization by male sperm (Figure 2B, WT). In contrast, mating with *uba-1(it129)* males produced an average of only 56 male vs. 236 hermaphrodite progeny (Figure 2B, *uba-1*), suggesting that the number of outcross progeny is reduced. The same data could be explained if the nullo-X sperm, which produce male progeny, are less competent for fertilization than the X-bearing sperm that produce hermaphrodites. This explanation seems unlikely, because the percentage of male progeny is elevated if the density of males for mating is increased (Figure 2B, *uba-1*, high #). To eliminate conclusively this possibility, *uba-1(it129)* males were mated to *fem-1(hc17)* adult hermaphrodites that lack sperm. Only outcross progeny are produced in this experiment and, although the numbers were low, males and hermaphrodites were observed in a ratio of 1∶1 (data not shown). Therefore, the protruding spicule phenotype observed in *uba-1(it129)* males at the permissive temperature decreases the successful transfer of sperm for fertilization.

Reproductive success was also characterized in the same manner for *uba-1(it129)* males shifted to the restrictive temperature at L3. No outcross progeny were observed from matings to either wild-type or *fem-1(hc17)* hermaphrodites. This failure might arise from the inability of sperm to fertilize the oocytes (as is true for hermaphrodite sperm at 25°C), or might be a consequence of the severe morphological defects in the male copulatory apparatus that occur at the restrictive temperature. A direct assessment of sperm transfer was performed to discriminate between the two possibilities. Males from *him-5(e1490)* strains that are wild-type or mutant for *uba-1(it129)* were raised at both 15°C and 25°C, stained with a fluorescent dye, then mated to *fem-1(hc17)* hermaphrodites that lacked sperm. Wild-type males reared at either temperature and mutant males reared at 15°C were successful in mating 50–70% of the time, as revealed by the presence of labeled sperm in the *fem-1(hc17)* hermaphrodites (Figure 2C, first two panels). In contrast, *uba-1(it129)* males raised at 25°C successfully transferred sperm in only two out of 10 instances. Although the efficiency of mating is reduced at 25°C, defects in the male copulatory structures arising from the *uba-1(it129)* mutation do not completely abrogate sperm transfer (Figure 2C, third panel). However, even those relatively rare successful matings do not give rise to outcross progeny, indicating that *uba-1(it129)* sperm from males are incapable of fertilization at the restrictive temperature.

An additional, sex-specific phenotype was observed *in uba-1(it129)* males: a late onset, progressive paralysis in two-thirds of the animals (Figure 2, D–F). The paralysis initiates at the posterior of the male and proceeds anteriorly as the worm ages, culminating in a completely paralyzed animal with a significantly shortened lifespan. Progressive paralysis is restricted to males, as *uba-1(it129)* hermaphrodites exhibit normal motility and lifespan (Figure 2F). The phenotype is not a consequence of aberrant somatic development but instead occurs post-developmentally, since delaying the temperature shift until adulthood still results in paralysis. Therefore, the *uba-1* gene product is required for the maintenance of neuromuscular function in the adult male.

### Sperm-Specific Defect of *uba-1* Mutation

Sperm development in *C. elegans* has been described in detail [39],[40], which allows the identification of specific cytological and functional defects in the developmental program that occur as a consequence of mutation. Normal spermatogenesis initiates from a mitotically dividing population of germ line stem cells. Primary spermatocytes separate from a syncytial cytoplasmic core and undergo a coordinated program of meiosis and differentiation. The two meiotic divisions give rise to four haploid spermatids with highly condensed nuclei. These small round cells separate from a larger residual body, which contains components not needed for subsequent steps in development. Activation by an extracellular signal converts the immotile spermatids into mature crawling spermatozoa capable of fertilization, and several compounds that promote activation *in vitro* have been identified [41]–[43]. Activation in hermaphrodites occurs in the spermatheca, where the mature spermatozoa are stored. Activation of male spermatids occurs at the time of insemination, and the male spermatozoa crawl from the uterus into the spermatheca. Fertilization takes place within the spermatheca as the oocyte squeezes into this chamber of hermaphrodite reproductive tract, and the newly formed zygote then passes into the uterus. Most of the spermatozoa are dislodged and must crawl back into the spermatheca to await the next oocyte.

**Figure 3:**
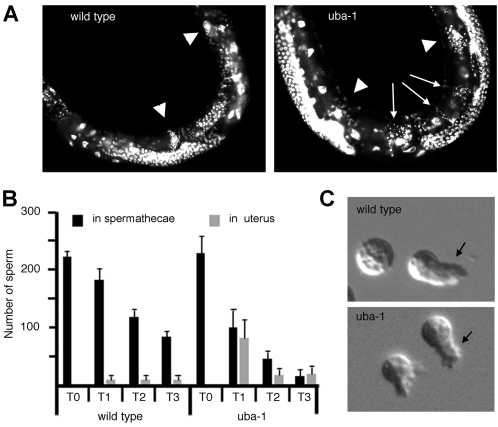
Sperm defects. A) Sperm localization in the hermaphrodite reproductive tract. Wild type and *uba-1(it129)* adults at 25°C were fixed and stained with DAPI to count sperm nuclei. Arrowheads, location of spermathecae; small arrows, sperm displaced into the uterus. B) Summary of sperm localization data. Shown are mean values and standard deviations per hermaphrodite (N = 6). T0, before egg-laying commences; T1, after 1–2 ovulations; T2, 8 h post-T1; T3, 8 h post-T2. C) *In vitro* activation. Spermatids from wild type and *uba-1(it129)* males at 25°C were activated with monensin. Arrow indicates pseudopod of crawling spermatozoon.

Sperm-specific sterility caused by the *uba-1(it129)* mutation was characterized in greater detail, beginning with the early events leading to spermatid formation. DAPI staining of L4 and young adult hermaphrodites and males revealed no differences in meiotic progression, the number of sperm produced, or (for hermaphrodites) their initial localization to the spermathecae (Figure 3, A–B and data not shown). Activation of spermatids was normal *in vivo* and *in vitro* and produced crawling spermatozoa with no discernible defect in pseudopod movement or cell motility (Figure 3C). Since motility and localization appear normal and yet no zygotes are formed, the *uba-1(it129)* mutation produces mature spermatozoa that are nonetheless incapable of fertilization.

A secondary defect in sperm function was detected later in adult hermaphrodites. Spermatozoa are displaced from the spermatheca into the uterus by each passing oocyte, and must return to the spermatheca and await the next egg. Fertilization efficiency is essentially 100% in wild type animals, with nearly every sperm being utilized for reproduction [38]. Thus, the number of sperm in the spermatheca decreases in concordance with an increase in the number of progeny produced. Because *uba-1(it129)* spermatozoa are motile but incapable of fertilization, one might predict that numbers within the spermatheca would remain high throughout oocyte production. Instead, the opposite phenomenon was observed, as sperm counts declined more rapidly in *uba-1(it129)* hermaphrodites than in wild type (Figure 3B). Furthermore, significant numbers of spermatozoa were detected in the uterus instead of the spermatheca (Figure 3A, *uba-1*). These cells are swept from the spermatheca by the unfertilized oocyte but are unable to return, and instead are expelled through the vulva when oocytes are deposited. Thus, although sperm motility and localization initially appear normal, these processes are clearly impaired in older animals. This observation may indicate a defect in the maintenance of sperm quality over time, which adversely impacts either motility or sperm-spermatheca interaction.

### Identification of *it129* as *uba-1*

**Figure 4:**
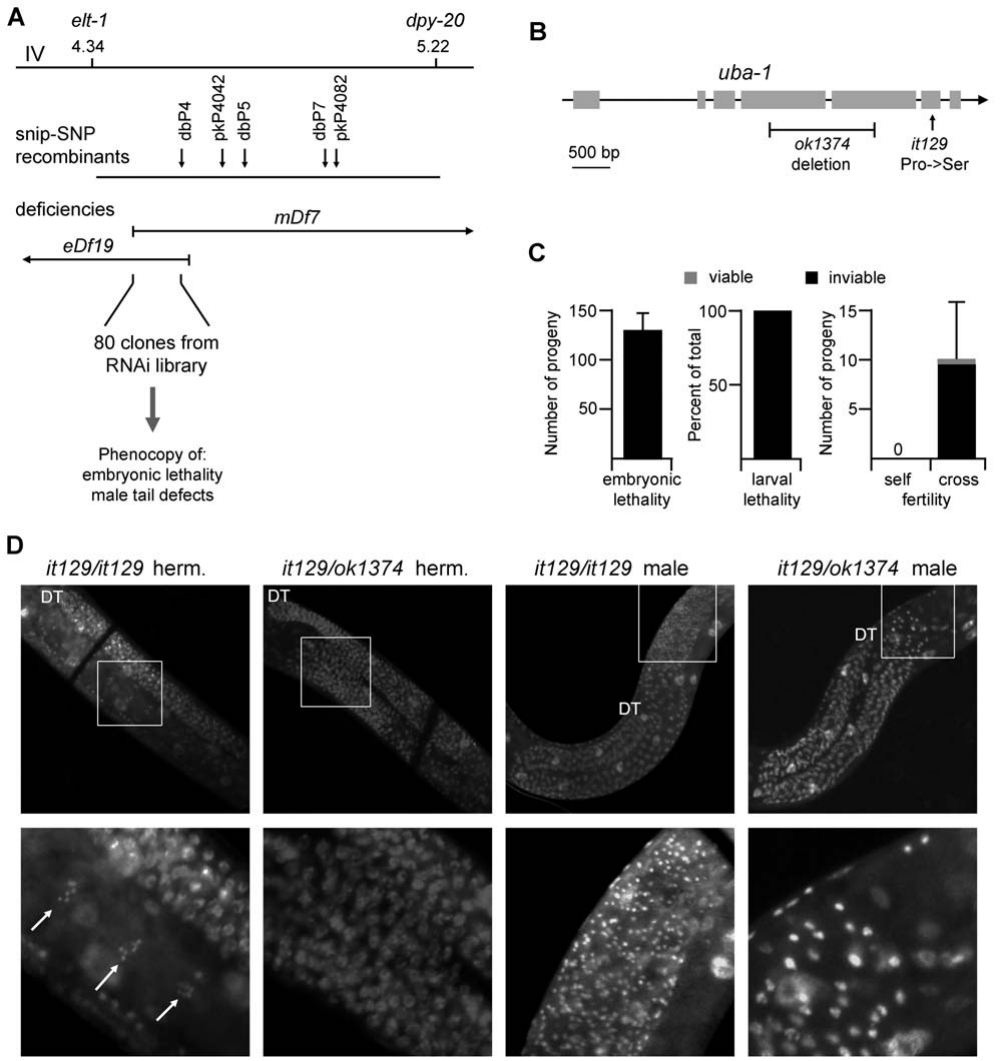
Cloning and complementation. A) Schematic of cloning strategy. Shown at top is the interval of chromosome IV from *elt-1* to *dpy-20*. Line two indicates the position of snip-SNPs identified in recombinant lines from N2 *uba-1(it129) dpy-20(e1282)* crossed with Hawaiian strain CB4856. The next two lines indicate the endpoints and overlapping regions of chromosomal deficiencies *eDf19* and *mDf7*, which failed to complement *uba-1(it129)*. Eighty genes within the 0.31 Mb overlap were screened by RNAi feeding for two *uba-1(it129)* phenotypes: embryonic lethality and male tail defects. B) Predicted gene structure of *uba-1*. Shown are position of the Pro1024Ser missense mutation identified in the *it129* allele and extent of the deleted region of the *ok1374* allele. C) Complementation data for *it129/ok1374* heterozygotes. Assay conditions for F1 embryonic lethality (N = 6 hermaphrodites), larval lethality (minimum 500 embryos), and sperm-specific sterility (N = 10 hermaphrodites) were identical to those used to characterize *it129* homozyogotes; see Figure 1 and accompanying text. D) Germ line defects in *it129/ok1374* heterozyogotes. Germ line nuclei were visualized by DAPI staining of fixed adult animals. The distal tip (DT) of the gonad is indicated for orientation. Top row shows a single gonad arm from (left to right) an *it129* homozygous hermaphrodite, *it129/ok1374* heterozygous hermaphrodite, *it129* homozygous male, and *it129/ok1374* heterozygous male. Bottom row shows a high-magnification image of the boxed region of the proximal gonad. Arrows, oocyte nuclei in diakinesis.

The identity of the *it129* allele was determined through a combination of genetic and physical mapping strategies (Figure 4A). Three-factor crosses placed this allele on chromosome IV between *elt-1* and *dpy-20* (F. Fell and S. Ward, pers. comm., and our own results; mapping data available at www.wormbase.org). Single nucleotide polymorphisms that overlap restriction sites (snip-SNPs) were analyzed in recombinant lines [44]. Strains containing the deficiency *eDf19* or *mDf7* failed to complement *it129*, further limiting its position to the overlapping 310 kb interval. A total of 80 candidate genes within the interval were available from a large-scale RNAi feeding library [35]. All were tested for the ability to replicate two of the *it129* phenotypes: F1 embryonic lethality of treated adult hermaphrodites, and tail deformation in adult males treated as larvae. Only one of the plasmids tested reproduced both phenotypes. That plasmid contains a fragment of the gene encoding the ubiquitin-activating enzyme E1, which in *C. elegans* is known as *uba-1*.

Complementation tests confirmed that *it129* is an allele of *uba-1*. The Gene Knockout Consortium (http://celeganskoconsortium.omrf.org) has generated a deletion allele, *uba-1(ok1374)*, that removes much of the third and fourth exons and is predicted to be a null mutation (Figure 4B). Mutants homozygous for *uba-1(ok1374)* exhibit embryonic lethality or early larval arrest, so *ok1374/it129* animals were obtained from crosses at the permissive temperature to allow recovery of viable lines. Complementation between the two alleles was tested by temperature shift at various developmental stages as described above. The identical phenotypes reported for *it129* homozygotes were observed for *ok1374/it129* heterozygotes: embryonic lethality, larval lethality, sperm-specific sterility, defects in male tail formation, and male-specific progressive paralysis (Figure 4C, and data not shown). Thus, *ok1374* and *it129* fail to complement each other and are both alleles of *uba-1*.

The *ok1374* deletion is a putative null allele, while the *it129* mutation is probably hypomorphic (i.e., reduction of function; see Discussion). Therefore, we sought to ascertain whether *it129/ok1374* heterozygotes were more adversely affected than *it129* homozygotes. Most of the phenotypes observed in the *it129* homozygous animals are highly penetrant, making enhancement difficult to detect. However, data from the complementation assay for sperm-specific sterility strongly suggest a more severe defect in *it129/ok1374* animals. Cross-fertilization of sterile *it129* homozygous hermaphrodites by wild type males yields progeny with high viability (96%; see Figure 1C). In contrast, cross-fertilization of sterile *it129/ok1374* hermaphrodites produces embryos with very low viability (6%; Figure 4C). Furthermore, the same data demonstrate that the number of fertilized embryos is significantly lower for *it129/ok1374* heterozygotes than *it129* homozygotes (10 vs. 48, respectively). Sperm are normally the limiting gamete for fertilization in *C. elegans*, but these results suggest that oocyte production might be defective in *it129/ok1374* hermaphrodites. Therefore, we examined the gonads of these strains directly by DAPI staining.

Germ cell development in *C. elegans* proceeds distally to proximally within the gonad, and is most readily distinguishable by changes in nuclear morphology [45]. In hermaphrodites, the proximal arm of the wild-type adult gonad contains a row of individual oocytes whose nuclei are arrested at diakinesis of meiosis I. Our analysis indicates that the germ lines of *it129* homozygotes are similar to wild type hermaphrodites, and the proximal gonad contains morphologically normal oocytes whose six diakinetic bivalents are easily seen (Figure 4D, top and bottom left panels). In contrast, the germ lines of *it129/ok1374* animals show an increased population of germ cells and a concommitant reduction in the number of oocytes in the proximal arm of the gonad. This defect in oogenesis is variable; some germ lines appear largely normal, while in other examples oocytes are absent and have been completely supplanted by an excess number of germ cells (as in Figure 4D, top and bottom second panels). A similar phenotype has been reported for mutations in a number of genes that govern the proliferation vs. meiosis decision, such as *glp-1* [46].

In addition, we also observed a spermatogenesis defect in the germ line of males. Wild-type adult males accumulate large numbers of highly condensed spermatid nuclei within the seminal vesicle. The *it129* homozygous males likewise contain an abundance of compact spermatid nuclei (Figure 4D, top and bottom third panels). However, the seminal vesicle of *it129/ok1374* males contain relatively few nuclei that also appear larger or less condensed than spermatid nuclei (Figure 4D, rightmost top and bottom panels). In both hermaphrodites and males, the mitotic and pachytene regions of the germ line in the distal gonad appear normal (albeit occasionally reduced in size). These results suggest that the differentiation of gametes in both sexes is impaired in the *it129/ok1374* heterozygous mutants, but with opposite effects depending upon the type of gamete: males possess fewer spermatids than normal, while hermaphrodites contain an excess of germ cell nuclei rather than oocytes. Gamete-specific differences in proliferation and differentiation have been reported previously. For example, loss of *gld-1* causes germ line overproliferation only in hermaphrodites undergoing oogenesis [47], while loss of *puf-8* causes overproliferation only in sperm-producing germ lines [48].

Transgene rescue of *it129* with the wild type *uba-1* gene further confirmed its identification. Initial attempts at rescue by germ line microinjection indicated that worms might be exquisitely sensitive to the dosage of this gene. Control injections with the *rol-6* marker [49] produced numerous F1 rolling progeny with stable transmission in subsequent generations. In contrast, coinjection of *uba-1* with *rol-6* at typical concentrations resulted in low brood sizes with very few F1 rollers and no stably transmitting lines, suggestive of transgene toxicity. To reduce the gene dosage, the concentration of *uba-1* DNA was decreased relative to *rol-6* and genomic N2 DNA was also included in the injections. At the lowest concentration tested, four of sixteen stably transmitting lines exhibited partial rescue of both sperm-specific sterility and embryonic lethality at the restrictive temperature. Therefore, the wild type *uba-1* transgene is able to complement the *it129* mutation.

**Figure 5:**
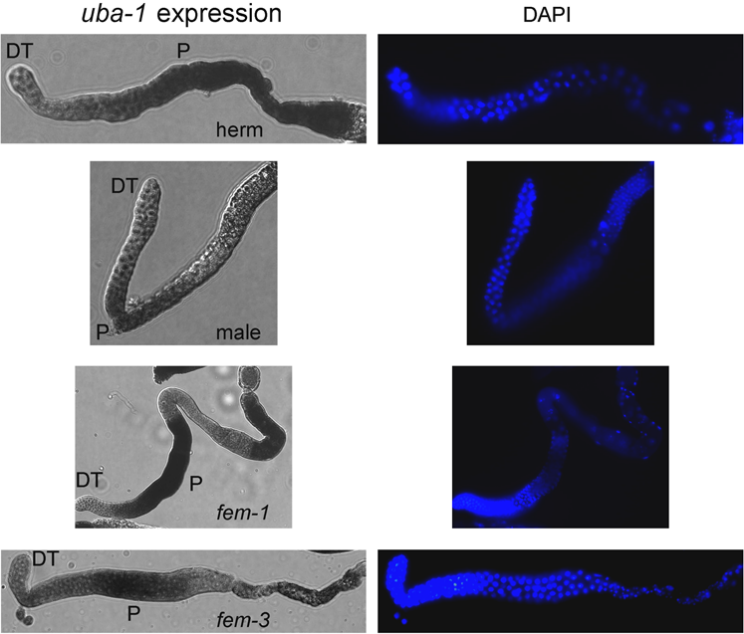
*In situ* hybridization of gonads. Panels on the left show the *uba-1* expression pattern in dissected gonads. Panels on the right show nuclear morphology by DAPI staining. From top to bottom, gonads are from adult hermaphrodites during oogenesis, adult males during spermatogenesis, *fem-1(hc17)* adult hermaphrodites that produce only oocytes, and *fem-3(q23)* adult hermaphrodites that make only sperm. At least 20 gonads were examined for each genotype or sex. DT, distal tip of gonad; P, pachytene region.

Expression of a *uba-1::GFP* reporter transgene has been reported in a variety of somatic tissues but not the germ line [50], although a functional role for UBA-1 in this tissue is indicated by the mutant phenotype. Transgenes are often silenced within the germ line, so *in situ* hybridization was employed to detect transcription of the endogenous *uba-1* gene within the gonad. Abundant expression was detected in germ cells that had initiated meiosis in wild type hermaphrodites (during sperm and oocyte production) and males (Figure 5). Signal intensity appeared to be higher during oocyte production; this observation was confirmed by comparing *fem-1(hc17)* hermaphrodites, which make only oocytes, to *fem-3(q23)* hermaphrodites, which make only sperm. Peak expression occurs at pachytene of the first meiotic division, is absent immediately afterwards, and is detected again in late oogenesis. This pattern is more apparent in the *fem-1(hc17)* gonad, which is from an older adult than the wild type hermaphrodite.

Sequence determination of the *uba-1* coding region from the *it129*-bearing strain revealed the molecular lesion. A single nucleotide substitution was detected that converts the proline at position 1024 to serine (Pro1024Ser, Figure 4B). The complete structure of E1 ubiquitin-activating enzyme has not yet been determined, but X-ray crystal structures of the activating enzymes for ubiquitin-like proteins SUMO and NEDD8 are available [51],[52]. The proline residue that is mutated in *uba-1(it129)* maps near the active site where the ubiquitin moiety is predicted to be covalently attached to the E1 protein. On the basis of the structural data, the Pro1024Ser mutation might be expected to alter catalytic activity of the enzyme.

### *In vivo* Defects in Ubiquitination and Embryogenesis

**Figure 6:**
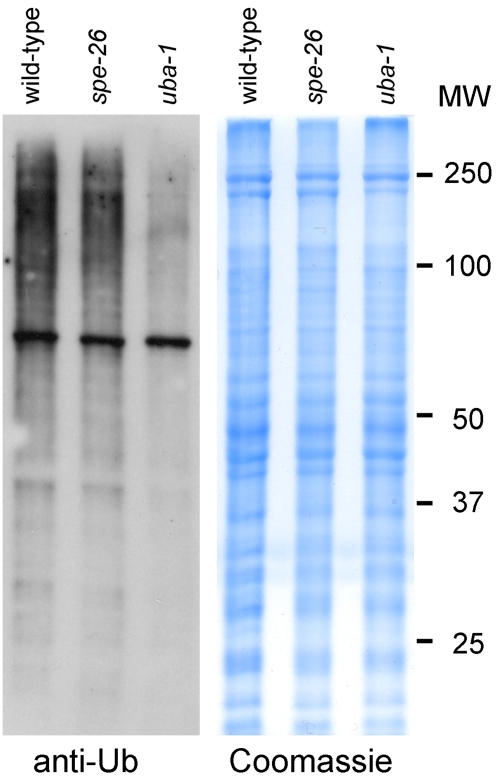
Western blot for ubiquitin. Panel on the left (anti-Ub) shows the overall level of ubiquitin conjugates from wild-type, *spe-26(it112)*, or *uba-1(it129)* young adult hermaphrodites. Equal amounts of soluble protein extracts were detected with ubiquitin-specific monoclonal antibody. Panel on the right (Coomassie) shows the same extracts stained for total protein. Size standards (MW) are indicated to the far right.

The *uba-1* gene encodes the only known E1 ubiquitin-activating enzyme in *C. elegans*, so a defect in its activity is predicted to impair subsequent steps in the enzymatic cascade and cause an overall decrease in the level of ubiquitination on substrate proteins. We tested this hypothesis directly by using ubiquitin-specific antibodies to assess the amount of ubiquitination in worm protein lysates. To control for variations in ubiquitination activity at different stages of development, we extracted protein from age-synchronized young adult hermaphrodites shifted as L3 larvae. Since these *uba-1(it129)* animals are infertile due to sperm-specific sterility, we included as an additional control a strain containing *spe-26(it112)* (a temperature-sensitive, sperm-specific sterile mutation). Western blots show a significant reduction in the amount of ubiquitin signal in *uba-1(it129)* protein extracts compared to wild-type and *spe-26(it112)* controls (Figure 6). Note that the level of ubiquitin in the high molecular weight region of the blot is particularly diminished, presumably reflecting a substantial reduction in the amount of poly-ubiquitinated substrates. Therefore, the *uba-1(it129)* mutation exhibits an *in vivo* decrease in protein ubiquitination.

Reduced ubiquitination is predicted to adversely impact proteasomal degradation of target proteins. Well-characterized roles for ubiquitin-mediated proteolysis in *C. elegans* occur during the early events of embryogenesis. The anaphase-promoting complex (APC) is an E3 ligase that is required for degradation of the meiotic inhibitor securin [53]. Complete loss of APC activity results in metaphase arrest of the one-celled embryo [19].

The *uba-1(it129)* mutation does not produce the one-celled arrest caused by loss of APC activity, but instead mimics the multicellular embryonic lethality resulting from reduced APC function. This phenotype is produced by hypomorphic mutations in APC components or by synthetic interactions between some pairs of temperature-sensitive alleles (i.e., each single mutation has no effect at the permissive temperature, whereas the combination of both mutations causes maternal embryonic lethality) [29]. Since UBA-1 and APC function in the same enzymatic cascade, mutations in both might likewise exhibit a synthetic interaction. Therefore, we tested the *uba-1(it129)* allele in combination with APC components. Double mutants of *uba-1(it129)* with either the APC subunit *mat-3(or180)* [19] or the APC activator *fzy-1(h1983)* [53] resulted in maternal embryonic lethality at the permissive temperature (Table 3).

**Table 3:** Synthetic interactions between double mutants.

Genotype	+	*mat-3(or180)*	*fzy-1(h1983)*
+	WT	WT	WT
*uba-1(it129)*	WT	Mel	Mel

WT, wild type; Mel, maternal embryonic lethality. Data are from a minimum of ten hermaphrodites for each genotype reared at the permissive temperature of 15°C.

**Figure 7:**
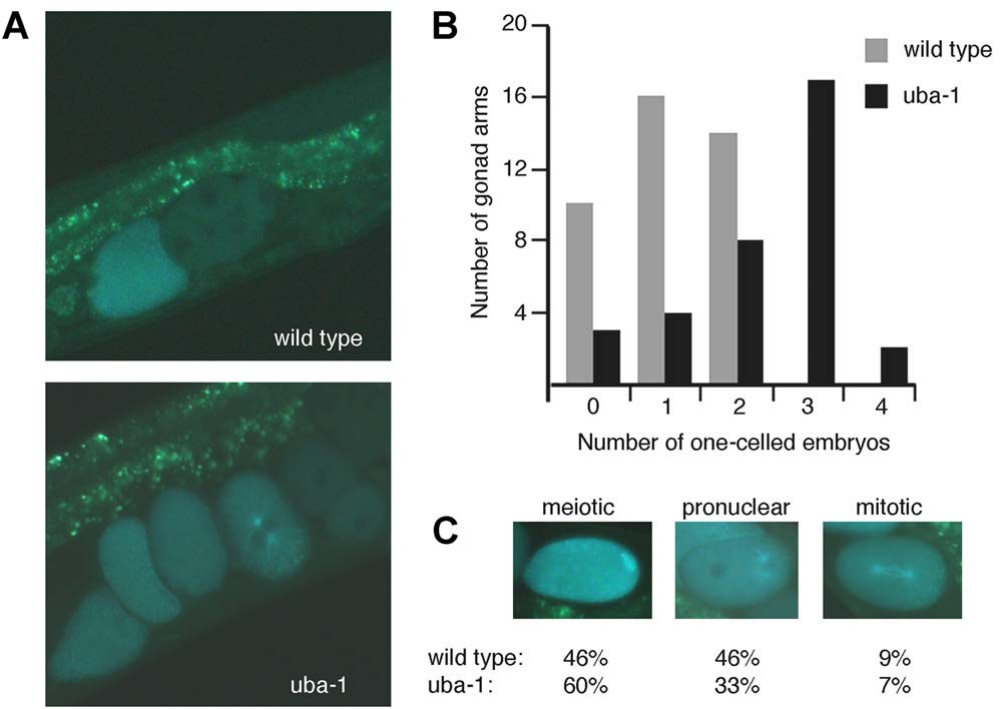
OMA-1::GFP expression. A) Adult hermaphrodites expressing the *oma-1::GFP* integrated transgene. Shown are examples of wild type and *uba-1(it129)* animals that contain one and four one-celled embryos, respectively. B) Frequency of one-celled embryos in the uterus. The number of one-celled embryos per gonad arm were counted for wild type (N = 40) and *uba-1(it129)* (N = 34) hermaphrodites. C) Distribution of one-celled embryos in the meiotic, pronuclear, and mitotic stages of development, as visualized by OMA-1::GFP.

Early embryogenesis was examined in *uba-1(it129)* adult hermaphrodites shifted to 25° for defects in meiotic progression or A-P polarity in the first cell division. An *oma-1::GFP* transgene was used to allow visualization of embryonic polarity [54]. In wild-type hermaphrodites, OMA-1::GFP protein is evenly distributed throughout the cytosol and excluded from the intact pronuclei of the one-celled embryo. Our observations at 25°C indicate that the protein is also concentrated on the sperm centrioles and mitotic spindle. Ubiquitin-mediated proteolysis at the first cell division degrades the bulk of OMA-1::GFP. The protein is absent in the anterior (A) cell of the two-celled embryo, and the remaining OMA-1::GFP becomes associated with P granules in the posterior (P) cell. In *uba-1(it129)* animals, the pattern of OMA-1::GFP in one-celled embryos is indistinguishable from wild-type. OMA-1::GFP degradation during the first cell division is likewise identical, and the protein persists only in the P cell. However, progression of the zygote through the first division is slower than normal for *uba-1(it129)* embryos. The delayed progression leads to an increase in the number of one-celled embryos within the uterus, which is easily visualized by the presence of OMA-1::GFP (Figure 7A). Wild-type hermaphrodites typically contain a single one-celled embryo in each arm of the gonad; in contrast, *uba-1(it129)* mutants possess an average of three one-celled embryos per gonad arm (Figure 7B). In addition, 35% of the *uba-1(it129)* hermaphrodites contained a crushed zygote within the uterus. Formation of the rigid eggshell is completed late in meiosis, so these crushed zygotes might be either an indirect consequence of the observed meiotic delay or indicate a structural requirement for ubiquitination in the embryo immediately following fertilization.

The delay in progression through the first embryonic division was examined in greater detail. Upon fertilization, the oocyte nucleus completes the first and second meiotic divisions. The oocyte and sperm pronuclei meet and fuse, then undergo the first mitotic division. The percentage of embryos observed at each of these stages (meiosis, pronuclear migration and fusion, and mitosis) was determined for wild-type and *uba-1(it129)* animals. The fraction of one-celled embryos in the meiotic and pronuclear stages was equivalent in wild type, but approximately two-fold higher in the meiotic stage for *uba-1(it129)* embryos (Figure 7C), suggesting that meiosis is acutely sensitive to UBA-1. Despite this significantly skewed distribution (p<0.001 by Pearson's chi-square test), there were no gross defects in nuclear or cellular morphology or OMA-1::GFP distribution as embryonic development progressed.

## Discussion

We report here the isolation and characterization of a temperature-sensitive mutation of the *uba-1* gene, which encodes the E1 ubiquitin-activating enzyme of *C. elegans*. Activation by E1 is the first step in the enzymatic pathway that leads to the conjugation of ubiquitin to target proteins. Manipulation of E1 activity by temperature shift provides a mechanism for identifying the many roles for ubiquitination throughout development. Effects of the *uba-1(it129)* mutation are manifested at both the organismal (i.e., embryonic and larval lethality, reduction in body size) and cellular (sperm-specific sterility) levels, and also result in sex-specific differences of developmental (formation of the male copulatory apparatus) and post-developmental (late-onset male paralysis) processes. The *uba-1(it129)* mutation causes a substantial reduction of *in vivo* levels of ubiquitin-conjugated substrates, exhibits synthetic embryonic lethality with components of the anaphase promoting complex (an E3 ubiquitin ligase), and produces delays in early embryonic events known to be regulated by ubiquitin-mediated proteolysis.

Taken together, the data indicate that the *uba-1(it129)* mutation results in a temperature-sensitive reduction in its ubiquitin-activating enzymatic activity. Since the *uba-1* gene product is the only E1 enzyme in *C. elegans*, a reduction in its activity is predicted to negatively impact the function of E2 and E3 enzymes globally. This reduction would extend the half-life of proteins normally targeted for the proteasome, as well as altering the localization and/or activities of other ubiquitin-conjugated substrates. Some of these downstream pathways will be more or less sensitive to a reduction in E1 activity, but the result will be a decrease in the rate of ubiquitination for a wide variety of substrate proteins. In support of this model, Western blotting with anti-ubiquitin antibodies demonstrated an overall reduction in ubiquitin labeling of extracts from the *uba-1(it129)* mutant strain (Figure 6). Also, structural data from related E1 enzymes predict that the Pro1024Ser mutation in *uba-1(it129)* might alter its catalytic activity. Finally, the model is consistent with our results in the candidate gene screen (in which reduced levels of UBA-1 by RNAi reproduced both the embryonic lethality and male tail defects) as well as the phenocopy of APC hypomorphic alleles rather than strong loss-of-function mutations (i.e., multicellular vs. one-celled embryonic arrest).

An alternative hypothesis, that the *uba-1(it129)* mutation blocks only one or a few E2/E3 pathways, is less likely. The observed reduction of *in vivo* ubiquitination in the mutant would require that the bulk of ubiquitin conjugation be mediated by one or a few E3 ligases; however, the hundreds of E3s that are present in *C. elegans* argue against this model. Furthermore, the range of phenotypes produced by the *uba-1* mutation is much broader than those reported for inactivation of any single E2 or E3 enzyme [55], consistent with its participation in multiple E2/E3 pathways. We clearly demonstrate genetic interactions between *uba-1(it129)* and one E3 pathway, the APC, via synthetic embryonic lethality with *mat-3* or *fzy-1* alleles. However, the sperm-specific fertilization defect appears to involve a different E3 pathway. This phenotype is not observed in APC mutants but has been reported for mutations in *spe-16*, which has recently been determined to encode an E3 ubiquitin ligase homolog (Steve L'Hernault, personal communication).

Some effects of the *uba-1* mutation can be interpreted in light of the variety of phenotypes that arise from the loss of individual E2 or E3 activities. For example, embryonic and larval lethality have been reported for a number of E2 and E3 homologs in large-scale RNAi screens [34]–[36]. However, the majority of these genes have not been further characterized and, absent additional knowledge of which proteins are substrates for particular E2 and E3 enzymes, it's difficult to speculate on the molecular mechanisms responsible for the observed lethality.

In other instances, the *uba-1* mutant phenotype suggests a previously unidentified role for ubiquitination. Body size in *C. elegans* is governed by a canonical TGF-ß signal transduction pathway that initiates with the DBL-1 ligand [56],[57]. Components of the TGF-ß pathway in other organisms are known to be regulated by ubiquitin conjugation [58]. Different ubiquitin modifications produce antagonistic effects on signal transduction: mono-ubiquitination of Co-Smad stabilizes the protein and promotes signaling, while poly-ubiquitination of R-Smad leads to its proteasomal degradation and down-regulation of signaling. Given the effects of the *uba-1* mutation on *C. elegans* body size, it seems likely that components of the DBL-1/TFG-ß pathway are similarly regulated by ubiquitin.

The sperm-specific sterility of *uba-1(it129)*, coupled with the recent identification of *spe-16* as an E3 ubiquitin ligase homolog (Steve L'Hernault, personal communication), indicate a previously uncharacterized role for ubiquitin in *C. elegans* spermatogenesis. Ubiquitination is known to be essential for sperm function in a wide variety of organisms, and roles in mammalian spermatogenesis include regulation of the meiotic cell cycle, histone modification and chromatin remodeling, protein sorting during sperm differentiation, and quality control for defective sperm [59]–[62]. In *C. elegans*, early events like meiosis appear unaffected by the *uba-1(it129)* mutation, suggesting that the infertility of these morphologically normal spermatozoa is due to a later defect in sperm development. In a manner analogous to mammalian sperm, ubiquitination in *C. elegans* might function in protein sorting as the spermatids divide from the residual body. Errors in this process are known to adversely affect sperm function: mutation of *spe-15*, which encodes a myosin homolog, impairs the asymmetric segregation of proteins during spermatid budding and causes sperm-specific sterility [63]. Alternatively, ubiquitination might promote proteasomal degradation of a protein that inhibits fertilization, and decreased activity of UBA-1 would lead to inappropriate persistence of the proposed inhibitor. Spermatid activation and downstream events occur in the absence of new protein synthesis, so degradation of pre-existing component(s) is a plausible mechanism of regulation. Another possibility is that *uba-1(it129)* infertility might reflect a role for ubiquitin-mediated proteolysis in the sperm-oocyte interaction. Fertilization in ascidians is mediated by an extracellular enzyme from sperm that conjugates ubiquitin to a sperm receptor on the egg surface, leading to its degradation via the proteasome [64]. Ongoing analysis is designed to determine if one (or more) of these hypotheses is correct.

Multiple E3 ligases are involved in formation of the reproductive structures of the male tail, so the defects observed in *uba-1* mutant males might arise from impairment of one or more known ubiquitination pathways. Mutation of *mat-1*, which encodes the CDC27 subunit of the APC, causes a diminution in the size of the fan and sensory rays similar to the defect produced by *uba-1(it129)* [29]. The heterochronic gene *lin-41*, which encodes a homolog of the RING finger subclass of E3 ligases, is also required for proper formation of the male tail. A decrease in LIN-41 function causes precocious retraction of the male tail so that the fan and rays are reduced or absent [65],[66]. The DBL-1/TGF-ß pathway (mentioned above) that determines body size also plays a role in formation of the spicules [67], and might be implicated in the protruding spicule phenotype of *uba-1(it129)* males.

The late-onset paralysis and associated lethality produced by the *uba-1(it129)* mutation is unusual in two regards: it is sex-specific, affecting only males, and can be induced after all somatic development is complete. There are few reports of such post-developmental phenotypes for *C. elegans*, and this property suggests a defect in the maintenance of neuronal and/or muscle function rather than its establishment. Roles for ubiquitination in *C. elegans* neuromuscular activity have been reported previously. Multiple E2 conjugating enzymes have been implicated in polyglutamine protein aggregation in muscle [68]. E3 ligase complexes that have been demonstrated to affect either muscle or neuronal function include CHN-1/UDF-2, APC, KEL-8/CUL-3, SCF/FSN-1/RPM-1, SCF/LIN-23, and SCF/SEL-10 [15], [16], [69]–[72]. However, the paralysis of *uba-1(it129)* males is distinct from the more subtle neuromuscular defects reported for other ubiquitin pathway components such as APC (decreased duration of forward movement) or KEL-8/CUL-3 (changes in nose touch response and spontaneous reversal frequency) [15],[16]. Furthermore, functional roles for all of these enzymes have been demonstrated in hermaphrodites, so the sex-specific ubiquitination that is responsible for male paralysis remains to be elucidated.

Why are male-specific processes, including the fertility defect of the male gamete (i.e., sperm), so acutely sensitive to the level of UBA-1 activity? One intriguing possibility involves the recently discovered role for ubiquitin-mediated proteolysis in the sex determination pathway. The TRA-1 transcription factor is the critical regulator of somatic and germ line sex determination and acts primarily as an inhibitor of male sexual fate [73]. Three FEM proteins negatively regulate TRA-1 activity and thereby promote male cell fates, including sperm development in hermaphrodites [74]. Starostina et al. [12] demonstrate that the FEM proteins form an E3 ubiquitin ligase complex with CUL-2 that binds to and promotes proteasome-dependent degradation of TRA-1. Impairment of UBA-1 function by mutation would be predicted to decrease activity of the FEM/CUL-2 E3 complex, leading to an increase in TRA-1 levels that would inhibit male developmental processes. This weakly feminizing effect might act synergistically with one or more of the E3 pathways described above. If this hypothesis is correct, then some of the sex-specific defects of the *uba-1* mutation might be suppressed by a decrease in TRA-1 activity.

The observation of synthetic embryonic lethality between *uba-1(it129)* and mutations in components of the APC suggests a powerful approach for identifying new functions for downstream components of the ubiquitin pathway. A number of E2 and E3 homologs exhibit detectable phenotypes in genome-scale RNAi screens, but the majority are indistinguishable from wild type [34],[35]. One possible explanation is that many of these enzymes are functionally redundant, and that the determination of their roles will require inactivation of multiple E2s or E3s. Alternatively, in some instances the reduction of E2 or E3 levels by RNAi might be insufficient to disrupt function. However, the *uba-1* mutation provides a sensitized genetic background for detecting decreased activity of downstream enzymes. Reanalysis by RNAi screening of the E2 and E3 homologs in the *uba-1* mutant strain is likely to reveal novel functions for a number of those genes whose roles are currently unknown.

## Materials and Methods

### Genetics

*C. elegans* strains were derived from the wild-type isolate N2 (Bristol) and contained one or more of the following mutations: *uba-1(it129)IV*, *uba-1(ok1374)IV*, *dpy-20(e1282)IV*, *fem-1(hc17)IV*, *fem-3(q20)IV*, *him-5(e1490)V*, *mat-3(or180)III*, *fzy-1(h1983)II*, *spe-26(it112)*, or chromosome IV deficiencies *eDf19* or *mDf7*. A linked *uba-1(it129) dpy-20(e1282)* double mutant strain was generated to facilitate discrimination of homozygous and heterozygous lines in some experiments. The integrated *oma-1::GFP* transgenic line was constructed by Reuyling Lin [54]. Strains were maintained on NGM plates seeded with *E. coli* strain OP50. Age-synchronized populations of embryos were obtained by sodium hypochlorite treatment of gravid hermaphrodites. Strains were maintained at 15°C and shifted to 25°C as indicated for phenotypic analysis. Genetic manipulations were carried out according to Brenner [75].

### Microscopy

Microscopy was performed with an Olympus BX51TF or Zeiss Axio Imager equipped with Nomarski DIC objectives and appropriate filter sets for fluorescent imaging and cooled CCD camera for image capture. Images were processed using the AxioVision (release 4.6) package and prepared for publication using Adobe Photoshop CS v. 9.0.2. Intact animals were typically mounted on 2% agarose pads for imaging. Body length was measured from captured images using ImageJ software v. 1.38.

### Sperm Assays

Sperm morphology was assessed by dissection of gonads from adult hermaphrodites or males in SM medium [43]. Nuclear DNA morphology was visualized by DAPI staining of sperm from dissected gonads. *In vitro* activation of male spermatids was by treatment with monensin on poly-lysine-coated slides [43]. Motility and localization of hermaphrodite sperm were determined in intact animals by fixation and staining with DAPI, then counting the number of sperm nuclei in the spermathecae and uterus. Sperm transfer was ascertained by vital staining of males [76] with the mitochondrial dye MitoTracker Red CMXRos (Molecular Probes), then mating to unstained hermaphrodites anesthetized with tricaine and tetramisole. After 12 or 24 h, fluorescently-labeled male sperm within the hermaphrodite reproductive tract were visualized by microscopy using rhodamine filters. Self-fertility of hermaphrodites was assessed by shifting individual L3 animals to 25°C and counting the entire brood size. Cross-fertility of males was determined by mating with individual wild-type hermaphrodites or *fem-1(hc17)* females, then counting the number of male and hermaphrodite progeny produced by each animal after mating.

### Cloning and Molecular Analysis

The *uba-1(it129)* mutation was localized to chromosome IV between *elt-1* and *dpy-20* by three-factor crosses. Single nucleotide polymorphisms that affect restriction sites (snip-SNPs) were employed as physical mapping markers of individual *uba-1(it129) dpy-20(e1282)* recombinants with Hawaiian strain CB4856 [44]. Deficiency mapping was performed by complementation testing in *uba-1(it129)/Df* heterozygous strains. RNAi of candidate genes was performed by feeding [77] and assessed by phenocopy of F1 embryonic lethality for treated adult hermaphrodites and by defects in adult tail morphology for treated L3 males. Complementation of the *uba-1(ok1374)* deletion allele was determined by generating *it129/ok1374* heterozygous animals and performing temperature-shift assays as described for phenotypic characterization. Transformation rescue [49] was obtained by germ line microinjection of a 6.0 kb genomic fragment of the wild-type *uba-1* gene mixed with plasmid pRF4, which contains the dominant roller marker *rol-6(su1006)*, and linearized N2 genomic DNA at concentrations of 1, 50, and 100 µg/ml, respectively. Stable roller transgenic lines were generated from *uba-1(it129)* hermaphrodites maintained at 15°C, then rescue of sperm-specific sterility and embryonic lethality was scored after shifting to 25°C.

The molecular lesion of the *uba-1(it129)* allele was identified by PCR amplification of the 6.0 Kb *uba-1* genomic interval from mutant worms followed by sequence determination. *In situ* hybridization for *uba-1* germ line expression was performed on dissected gonads following fixation [78]. Digoxigenin-labeled, single-stranded sense and antisense probes were generated from a 1 kb cDNA fragment by linear amplification according to the manufacturer's protocol (Roche, Indianapolis, IN). Following hybridization, probe detection was by colorimetric assay with alkaline phosphatase-conjugated anti-digoxigenin antibodies and NBT/BCIP substrate.

Western blot analysis was performed on soluble worm lysates from age-synchronized young adult hermaphrodites shifted to 25° as L2 larvae. Lysates were obtained by one freeze-thaw cycle, homogenization, and centrifugation for 10 minutes at 10,000 RCF. Protein concentration of the soluble fraction was quantified by Bradford assay. 10 µg samples were fractionated by SDS-PAGE and transferred to PVDF membranes. Ubiquitin-conjugated proteins were detected by mouse anti-ubiquitin monoclonal antibody (1°) followed by HRP-conjugated goat anti-mouse IgG polyclonal antibody (2°; both Stressgen, Ann Arbor, MI). Duplicate gels were stained with Gelcode Blue (Pierce, Rockford, IL) to visualize total protein.
